# Protective Effects of Xyloglucan in Association with the Polysaccharide Gelose in an Experimental Model of Gastroenteritis and Urinary Tract Infections

**DOI:** 10.3390/ijms19071844

**Published:** 2018-06-22

**Authors:** Emanuela Esposito, Michela Campolo, Giovanna Casili, Marika Lanza, Domenico Franco, Alessia Filippone, Alessio F. Peritore, Salvatore Cuzzocrea

**Affiliations:** 1 Department of Chemical, Biological, Pharmacological and Environmental Sciences, University of Messina, 31-98166 Messina, Italy; campolom@unime.it (M.C.); gcasili@unime.it (G.C.); mlanza@unime.it (M.L.); dfranco@unime.it (D.F.); afilippone@unime.it (A.F.); a.peritore2@campus.unimib.it (A.F.P.); 2 Department of Pharmacological and Physiological Sciences, Saint Louis University School of Medicine, St. Louis, MO 63104, USA

**Keywords:** xyloglucan, gelose, gastroenteritis, urinary tract infection

## Abstract

Acute infectious gastroenteritis (GE) and urinary tract infection (UTI) are common diseases and are normally perceived as mild and limiting illnesses. Xyloglucan is a natural plant polymer with protective barrier properties, also known as “mucosal protectors”, which is the main ingredient of medical devices developed for the management of different diseases, such as gastrointestinal diseases, urinary tract infections, or respiratory allergic diseases. The aim of this study was to evaluate the protective effect of xyloglucan in association with gelose (also called agar) in an experimental model of bacterial GE and UTI in rats. Two kinds of infection were induced by oral administration of *Salmonella enterica* and *Enterococcus hirae* for three days. Two days before the bacterial administration, preventive oral treatment with xyloglucan + gelose (10 mg/kg + 5 mg/kg) was performed daily until the seventh day. Twenty-four hours after the last treatment, rats were sacrificed and urinary tracts and intestines for different analysis were collected. The results showed that xyloglucan plus gelose was able to reduce intestinal morphological changes (*p* < 0.05 for both), tight junctions (TJ) permeability (*p* < 0.001 for both), and neutrophil infiltration (*p* < 0.05 for both) induced by bacterial infections, highlighting its barrier proprieties. Moreover, the compound reduced the number of bacterial colonies in the urinary tract favoring elimination by feces. The results obtained in the present study suggest that the protective barrier properties of xyloglucan plus gelose allow the prevention of GE and UTI in models of infections in rats.

## 1. Introduction

Acute infectious gastroenteritis (GE) and urinary tract infection (UTI) are common diseases and are normally perceived as mild and self-limiting. However, especially in vulnerable individuals such as smaller children, immuno-compromised persons, and the elderly, GE and UTI may lead to health care contacts and even hospitalization because of complications like sepsis or dehydration [1,2].

*Salmonella enterica* serovars Enteritidis and Typhimurium are involved in foodborne gastroenteritis throughout the world; therefore, to find appropriate measures to reduce salmonella contamination in the gastrointestinal and urinary tracts, it is essential to understand the mechanisms of salmonella infection, intestinal colonization, persistence, and excretion. During foodborne Salmonella enteritidis infections, pathogens can overpass the gut epithelial cell lining and translocate to extra-intestinal organs like the spleen, liver, and bladder [3]. In response to mucosal invasion, epithelial cells and macrophages express pro-inflammatory cytokines, e.g., interleukin-1β (IL-1β), to recruit neutrophils [4,5]. Moreover, although it is a rare cause, several authors have demonstrated a growing frequency of *Salmonella* spp. isolated in urine samples from UTIs with an unrecognized cause or related to immunodeficiency or a urological abnormality [6,7,8]. Over the past decade, *Enterococcus* spp. has been recognized as important pathogens which cause a wide spectrum of human infection, such as septicemia, urinary tract infections, and endocarditis. the attention has been focused on *Enterococcus hirae* (*E. hirae*), which is known to cause infection in animals such as rats and birds, but it is uncommonly encountered in clinical isolates from humans [9]. It has been shown that an oral inoculation of *E. hirae* could reproducibly cause diarrheal disease in infant rats, highlighting the significance of this group of organisms in the etiology of acute enteritis [10]. *E. hirae* has been described as an emergent nosocomial pathogen in hospital-acquired infections such as UTI. Despite only 3% of human infections detected in clinical practice from enterococcus is due to *E. hirae*, an emerging role in the onset of serious diseases (endocarditis, acute pancreatitis, pyelonephritis, and septic shock) can be attributed to this microorganism [11,12].

In the field of GE and UTI treatments, non-pharmacological oral supplements, including cranberry proanthocyanidins [13,14,15], probiotics [16], and mucosal protectors (such as xyloglucan) have been evaluated for the protection against these two diseases [17,18]. Xyloglucan belongs to a new class of products; it is extracted from the seeds of the tamarind tree (*Tamarindus indica*) and is defined as “mucosal protectors” which form a bio protective film, restoring the physiological functions of the intestinal walls. Results of recent clinical studies showed that the administration of xyloglucan is a fast, efficacious, and safe option for the treatment of acute diarrhea in adults and children [19,20,21]. Moreover in vitro studies supported the use of xyloglucan for the management of UTIs in routine clinical practice [22] and demonstrated that xyloglucan can prevent UTIs by reducing the intestinal reservoirs of uropathogenic strains (particularly *Escherichia coli*), interfering with the colonization of the perianal region and the urinary tract [17]. The aim of this study was to evaluate the protective effect of xyloglucan in association with gelose in gastrointestinal and urinary infections. Gelose (also called agar) is an easily melted biocompatible polysaccharide which contains a rich variety of essential elements of human body, like iodine, calcium, and magnesium. It has been largely used in the biomedical field due to its good biocompatibility and non-toxicity, showing a thermally reactive sol-gel transition property similar to gelatin. Regarding the biomedical applications of gelose, it has been developed as an agar granule that was used as a vehicle for the oral administration of a therapeutic agent [23,24]. Based upon this outstanding feature, its association with xyloglucan could have important beneficial effects on intestinal and bladder mucosa, favoring a longer xyloglucan intestinal and urinary bioavailability and prolonging its efficacy.

## 2. Results

### 2.1. Effect of Xyloglucan-Gelose on Intestinal Damage and Neutrophil Infiltration

**Figure 1:**
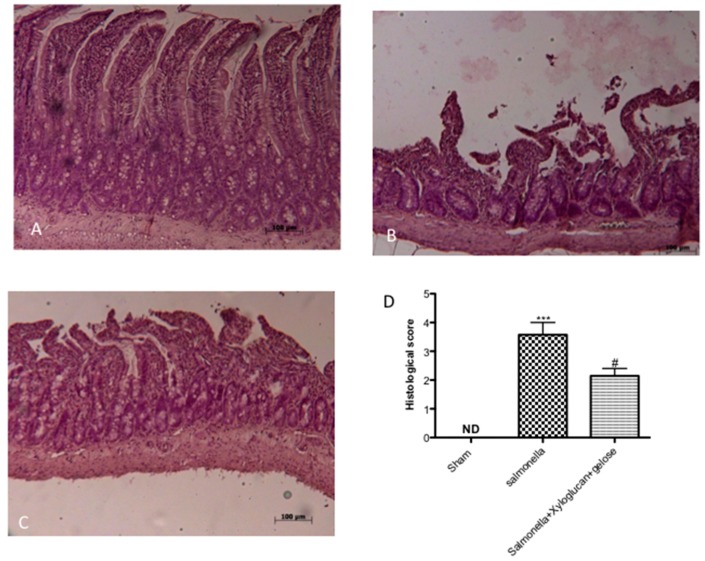
(**A**) The sham group; (**B**) Histological examination of intestine showed transmural necrosis, edema, and leukocyte infiltration in the submucosa of intestine following *S. enterica* inoculation compared to sham group; (**C**) Xyloglucan + gelose treatments significantly reduced the degree of tissue injury; and (**D**) The histological score. Values are means ± SEM of 10 animals for each group. *** *p* < 0.001 vs. Sham, ^#^ *p* < 0.05 vs. *S. enterica*.

**Figure 2:**
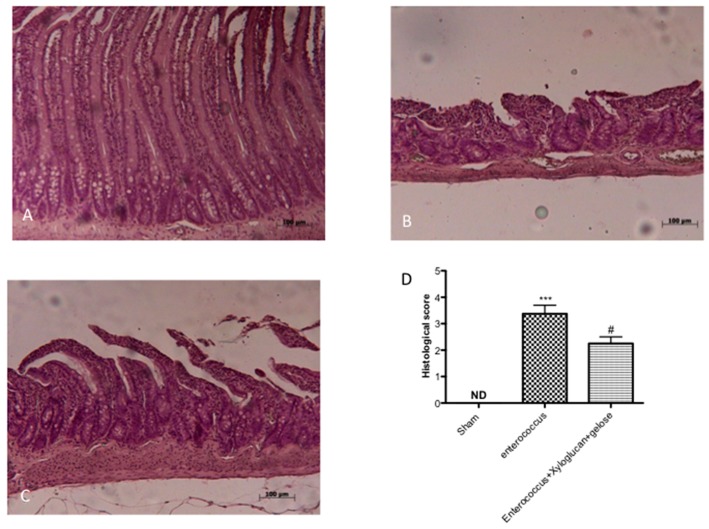
(**A**) The sham group; (**B**) Intestine from *E. hirae* inoculated rats showed transmural necrosis, edema, and leukocyte infiltration in the submucosa of tissue compared to the sham group; (**C**) Xyloglucan + gelose treatments appreciably reduced the degree of tissue injury; and (**D**) The histological score. Values are means ± SEM of 10 animals for each group. *** *p* < 0.001 vs. Sham, ^#^ *p* < 0.05 vs. *E. hirae*.

**Figure 3:**
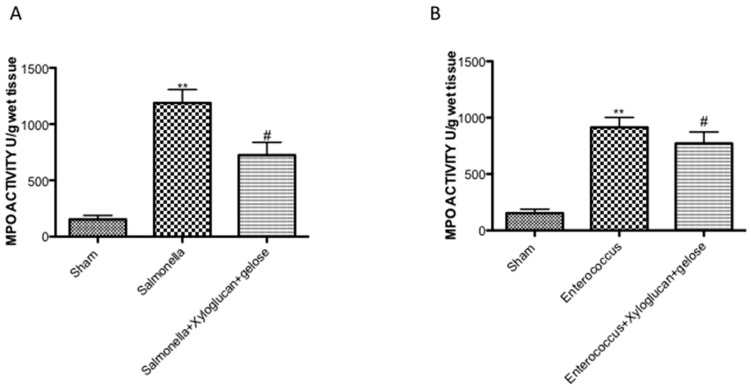
(**A**) MPO activity in intestine homogenates was significantly elevated from *S. enterica* inoculated rats. A significant decreasing in MPO activity was observed in intestine from rats treated with xyloglucan + gelose (10 mg/kg + 5 mg/kg); and (**B**) The same situation was observed for *E. hirae* inoculated rats. Pictures were captured at 10× magnification. Figures are representative of at least three separate experiments. Values are means ± SEM of 10 animals for each group. (**A**) ** *p* < 0.01 vs. Sham, ^#^ *p* < 0.05 vs. *S. enterica*. (**B**) ** *p* < 0.01 vs. Sham and ^#^ *p* < 0.05 vs. *E. hirae.*

The histological examination of the intestine revealed characteristic pathological changes following *S. enterica* and *E. hirae* infections compared to the sham group (Figure 1B, Figure 2B, respectively). The histopathological features included a transmural necrosis and edema and a diffuse leukocyte cellular infiltrate in the submucosa of intestine section from both *S. enterica* and *E. hirae*-infected rats compared to the sham groups (Figure 1A, Figure 2A, respectively). Xyloglucan + gelose treatments significantly reduced the degree of tissue damage following both infections (Figure 1C, Figure 2C, respectively). The histological scores were made by an independent observer (Figure 1D, Figure 2D, respectively). The myeloperoxidase (MPO) activity (index of polymorfonucleate infiltration) in intestine homogenates from *S. enterica* or *E. hirae*-inoculated rats was significantly elevated (Figure 3A,B, respectively). A decrease in MPO activity was observed in the intestines of rats treated with xyloglucan + gelose (10 mg/kg + 5 mg/kg) (Figure 3A,B, respectively).

### 2.2. Effect of Xyloglucan-Gelose on TJ Permeability

**Figure 4:**
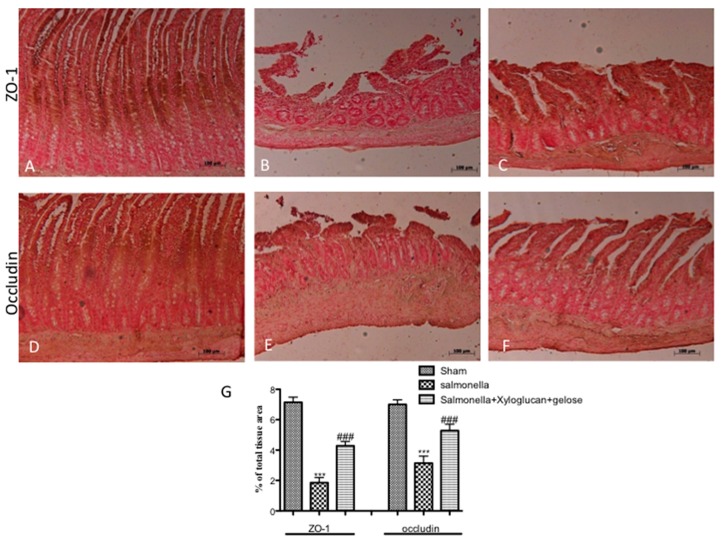
Immunostaining for ZO-1 and occludin expression in sham mice ((**A**) and (**D**) respectively); positive staining for ZO-1 and occludin following *S. enterica* infection ((**B**) and (**E**) respectively) and mice treated with xyloglucan + gelose ((**C**) and (**F**) respectively). (**G**) Densitometric analysis. Data are representative of at least three separate experiments. Images are representative of all animals in each group. Pictures were captured at 10× magnification (scale bar = 100 μm). Figures are representative of at least three separate experiments. Values are means ± SEM of 10 animals for each group. (ZO-1). *** *p* < 0.01 vs. Sham, ^###^ *p* < 0.001 vs. *S. enterica*; (occludin) *** *p* < 0.001 vs. Sham and ^###^ *p* < 0.001 vs. *S. enterica.*

**Figure 5:**
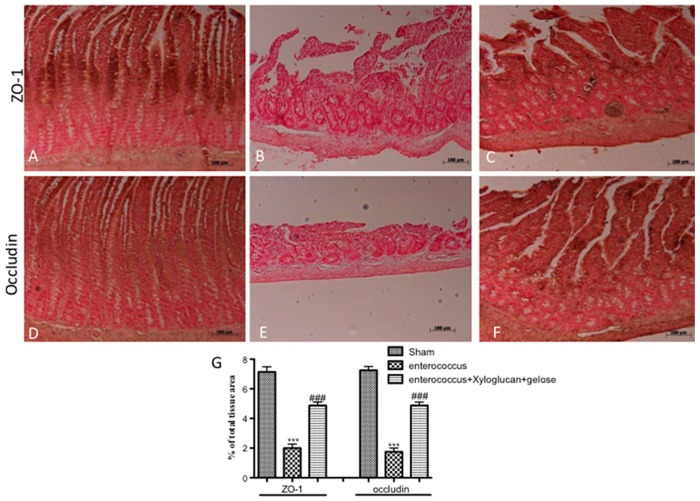
Immunostaining for ZO-1 and occludin expression in sham mice ((**A**) and (**D**) respectively). Positive staining for ZO-1 and occludin following *E. hirae* infection ((**B**) and (**E**) respectively) and mice treated with xyloglucan + gelose ((**C**) and (**F**) respectively). (**G**) Densitometric analysis. Data are representative of at least three separate experiments. Images are representative of all animals in each group. Pictures were captured at 10× magnification (scale bar = 100 μm). Values are means ± SEM of 10 animals for each group. Figures are representative of at least three separate experiments. (ZO-1) *** *p* < 0.01 vs. Sham, ^###^ *p* < 0.001 vs. *E. hirae*; (occludin) *** *p* < 0.001 vs. Sham and ^###^ *p* < 0.001 vs. *E. hirae.*

*S. enterica or E. hirae* inoculation induced an increase of TJ permeability throughout the entire small intestine, and the extent of alterations correlates with colonic damage (Salmonella and Enterococcus panels in Figure 4 and Figure 5) compared to sham groups (sham panels in Figure 4 and Figure 5). On the contrary, a significant reduction of zonula occludens-1 (ZO-1) and occludin positive staining was observed in xyloglucan + gelose (10 mg/kg + 5 mg/kg) treated rats (Salmonella + xyloglucan + gelose and Enterococcus + xyloglucan + gelose panels in Figure 4 and Figure 5). Densitometric analyses are showed in Figure 4A, Figure 5A respectively.

### 2.3. Bactericidal or Bacteriostatic Effect of Xyloglucan-Gelose

Twenty-four hours after inoculation, an *S. enterica* or *E. hirae* growth of >10^6^ colony forming units (CFU)/mL was found on the agar plate. This result indicated that xyloglucan-gelose has no direct toxic effect on *S. enterica* or *E. hirae*, so we can consider this association not bactericidal or bacteriostatic.

### 2.4. Effect of Xyloglucan-Gelose on Urine Volume and pH

**Figure 6:**
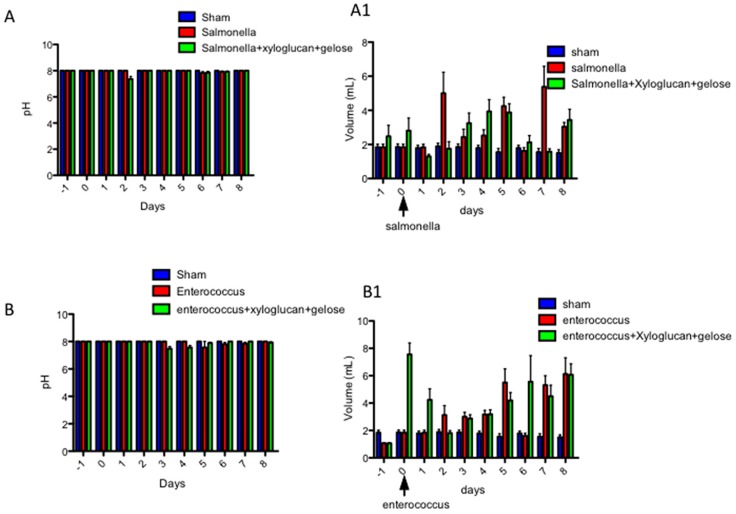
(**A**,**B**) Urinary pH values were recorded every day for eight days; and (**A1**,**B1**) Urine volume was collected every day for eight days. Values are means ± SEM of 10 animals for each group. Figures are representative of at least three separate experiments.

Urinary pH values were recorded every day for eight days. Our data showed that the pretreatment with xyloglucan + gelose did not significantly modify pH values compared to bacterial infection (Figure 6A,B). Moreover, urine volume was significantly increased following treatment with xyloglucan + gelose (10 mg/kg + 5 mg/kg) at 3 days after *S. enterica* (3.25 vs. 1.85) and 2 days after *E. hirae* inoculation (4.23 vs. 1.83) (Figure 6A1,B1).

### 2.5. Effect of Xyloglucan-Gelose on Bacterial Infection of the Urinary Tract

**Figure 7:**
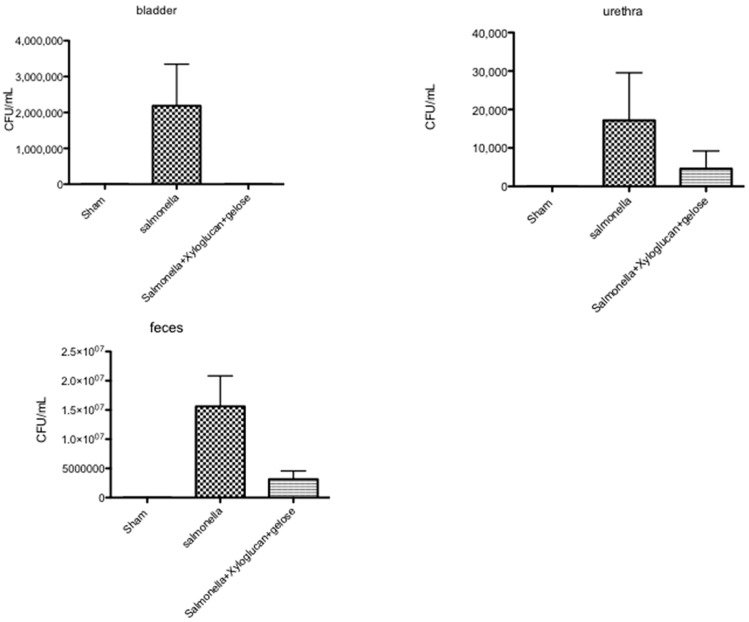
The number of bacteria recovered from bladder, urethra, and feces of rats killed eight days after *S. enterica inoculation*. Seven days after *S. enterica* inoculation, all rats had positive bladder and urethra cultures (bladder and urethra graphs). The number of bacteria recovered from the bladder showed a tendency to decrease in both the bladder and urethra after xyloglucan + gelose treatment (bladder and urethra graphs). *S. enterica* was significantly eliminated from the urinary tract but not from feces following xyloglucan + gelose treatments (feces graphs). Figures are representative of at least three separate experiments. Values are means ± SEM of 10 animals for each group.

**Figure 8:**
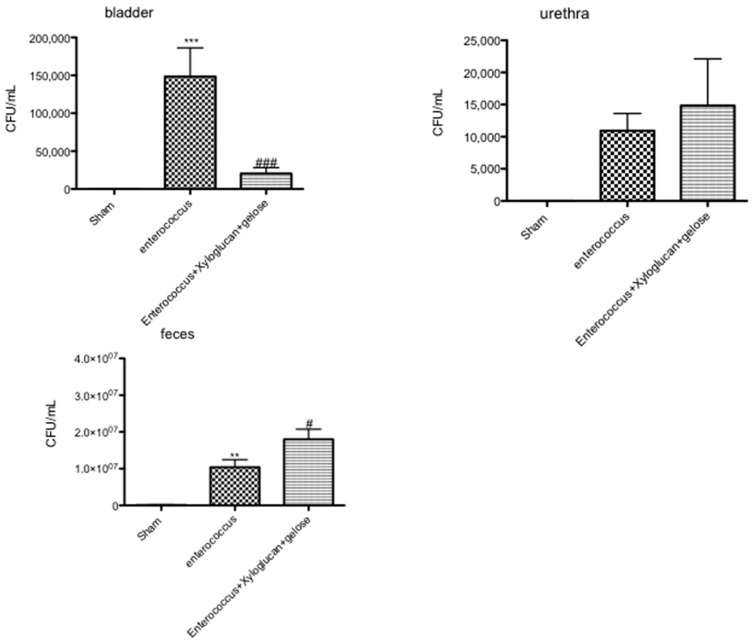
Rats infected with *E. hirae* showed positive bladder and urethra cultures while the treatment with xyloglucan + gelose significantly reduced the bacteria titers in the bladder but not in urethra (bladder and urethra graphs). Moreover, following xyloglucan + gelose treatment, *E. hirae* was significantly eliminated from the feces (feces graphs). Values are means ± SEM of 10 animals for each group. Figures are representative of at least three separate experiments. (Bladder) *** *p* < 0.01 vs. Sham, ^###^ *p* < 0.001 vs. *E. hirae*; (feces) ** *p* < 0.01 vs. Sham and ^#^ *p* < 0.05 vs. *E. hirae.*

The number of bacteria recovered from the bladder, urethra, and feces of rats killed eight days after inoculation are shown in Figure 7 and Figure 8. Seven days after *S. enterica* inoculation, all rats had positive bladder and urethra cultures. The number of bacteria recovered from the bladder showed a tendency to decrease in both the bladder and urethra after xyloglucan + gelose oral treatment. In contrast, *S. enterica* was eliminated rapidly and significantly from the urinary tract but not from the feces following xyloglucan + gelose treatments (Figure 7).

Rats infected with *E. hirae* showed positive bladder and urethra cultures, while the treatment with xyloglucan + gelose significantly reduced the bacteria titers in the bladder but not in the urethra. Moreover, following xyloglucan + gelose treatment, *E. hirae* was significantly eliminated from the feces (Figure 8).

## 3. Discussion

In the context of increasing antibiotic resistance, products that can prevent infectious diseases are of great interest [16].

A new class of compounds defined as “mucosal protectors”, is currently being used in the management of gastrointestinal diseases. The peculiarity of these products is the formation of a bioprotective film on the intestinal mucosa, preventing contact with pathogens and their products such as toxins, lipopolysaccharide (LPS), etc.; improving the resistance to pathologic aggression; and helping to restore normal mucosal function [25,26]. Among these film-forming products, gelatin tannate, gelatin, and xyloglucan are currently being used for gastroenteric disorders [25,26,27].

It has been shown that xyloglucan, in appropriate amounts, was able to form a protective biofilm on the intestinal mucosa [21]; in particular, xyloglucan has been proposed as a medical device for the management of UTI and GE by avoiding bacterial contact and reducing the intestinal reservoirs of uropathogenic strains, thus preventing contact with the urethra [17,22].

On the basis of xyloglucan properties, the aim of our study was to associate this compound with polysaccharide gelose, in order to favor longer xyloglucan bioavailability in the intestine and bladder.

Bacterial gastroenteritis is a self-limiting disease typical in developing and developed worlds. The identification of an etiological agent for bacterial gastroenteritis is possible through an identification of bacterial stool culture for the management of patients with severe or prolonged diarrhea or a history that may predict a complicated course of disease. *S. enterica* causes a self-limiting GE in humans, and such bacteria generally do not disseminate beyond the lamina propria and gut-associated lymphoid tissue [28,29]. *S. enterica* generally do not develop intestinal inflammation in mice but they cause systemic inflammation [30]. However, systemic infection is always accompanied by acute intestinal inflammation, which has very similar symptoms observed in human non-typhoidal Salmonella-mediated GE: the intestine shows polymorph nuclearneutrophilic infiltration (PMN), epithelial destruction, and crypt abscesses [28,30]. In fact, our results clearly showed significant intestinal morphological changes following Salmonella oral infection, accompanied by an increasing of MPO production by neutrophils. Conversely, the treatment with xyloglucan plus gelose prevented the onset of edema formation, villi disruption, and neutrophil infiltration. The intestinal epithelium functions as a physical barrier. Tight junctions seal epithelial cell layers, performing a gate: function that limits paracellular passage of water, ions, solutes, and immune cells. Moreover, TJ perform a “fence” function, regulating cell polarity by acting as diffusion barriers that materially separate apical and basolateral membrane components. Infection by *S. Typhimurium* causes a fast and progressive increase in the permeability of polarized epithelial monolayers and induces alterations in the localization of the TJ-associated proteins ZO-1 and occludin [31,32,33]. TJ disruption likely represents an important aspect of *S. enteritis* and may be central to the clinical manifestation of diarrhea [34,35]. Our data confirmed this dysregulation; however, a significant reduction of TJ permeability was seen following xyloglucan plus gelose treatment.

Enterococci are not usually considered to be gastrointestinal pathogens and, when isolated, are usually assumed to be normal intestinal flora unrelated to the diarrheal illness. However, in recent years, enterococci have been associated as a cause of diarrhea in animals. Different studies have shown that *E. hirae* was found in a diarrheic foal [36]; moreover, this isolate also causes diarrhea when inoculated into gnotobiotic piglets [36]. Additionally, a second isolate of *E. hirae* has been associated with diarrhea in suckling rats [37]. In all of these occasions, the enterococci coated the brush border of the small intestinal villi. Our results clearly showed an alteration of intestine architecture following *E. hirae* oral infection, accompanied by an increasing neutrophil infiltration. Moreover, the infection with *E. hirae* induced an increasing of TJ permeability, accompanied by a decreasing of ZO-1 and occludin positive staining. However, the association between xyloglucan and gelose was able to reduce all histological and molecular changes typical of enterococcus infection.

During foodborne *S. enterica* and *E. hirae* infection, pathogens can pass the gut epithelial cell lining and translocate to extra-intestinal organs like the spleen, liver, and bladder [3]. Moreover, it has been shown that both bacteria are models of UTIs related to gastrointestinal infections and other physiological disorders [3,6,7,11,12]. Therefore, our attention was focused on the effect of xyloglucan plus gelose following salmonella and enterococcus oral infection in the bladder.

It is well known that urinary volume may be related to the development of urinary tract infections. Our data showed a clear difficulty for animals in urinating following an infection: this leads to a reduction in urinary volume. Moreover, the urethra and bladder of *S. enterica* and *E. hirae* infected rats showed positive culture for both organs. The treatment with xyloglucan plus gelose showed important effects by increasing urine volume and decreasing bacterial growth in the urinary tract. In addition, the association was able to rapidly eliminate bacteria from feces. This can be explained considering the different strategies of invasion of bacteria, namely ascending and hematogenous pathways. About *E. hirae*, urethra infection can be due to ascending infection of uropathogens from the fecal flora to urinary tract. On the other hand, *S. enterica* infection occurs via the hematogenous route. Therefore, the presence of xyloglucan could create a bio-protective barrier to avoid the contact of the uropathogenic strain on the uroepithelium, favoring the elimination of bacteria from the urinary tract. These findings show that xyloglucan, in association with gelose, offers a novel modality to protect intestinal and bladder epithelium during infection. In particular, its efficacy is related to the modulation of TJ and neutrophil infiltration in the intestine. Furthermore, it can reduce the number of bacterial colonies in the urinary tract favoring elimination by feces. In conclusion, we have demonstrated the non-pharmacological barrier properties of xyloglucan and gelose on intestinal and uroepithelium infection, thus confirming the role of these compounds for the management of a GE and UTIs-infection model.

## 4. Materials and Methods

### 4.1. Materials

All chemicals were of the highest commercial grade available. All stock solutions were made in non-pyrogenic saline (0.9% NaCl, Baxter, Milan, Italy) or 10% dimethyl sulfoxide (Gibco, Thermo Fisher Scientific, Rodano, Italy). Xyloglucan + gelose were kindly provided by Novintethical Pharma SA (Lugano, Switzerland) and diluted in saline.

### 4.2. Animals

Sprague–Dawley male rats (200–230 g, Envigo, Udine, Italy) were used for the study. Food and water were available ad libitum. The animals were fed with a standard diet. The University of Messina Review Board approved the study, in agreement with Italian regulations on protection of animals used for experimental purposes (Ministerial Decree 650/2017-PR of 21/08/2017). Animal care agreed with Italian regulations on the protection of animals used for experimental and other scientific purposes (D.M.116192) as well as with the EEC regulations (O.J. of E.C. L 358/1 12/18/1986). This study conforms to the “ARRIVE Guidelines for Reporting Animal Research”. Authors declare that the research complies with the commonly-accepted “3Rs”: Replacement, Reduction and Refinement.

### 4.3. Salmonella enterica and Enterococcus hirae Infection

**Figure 9:**
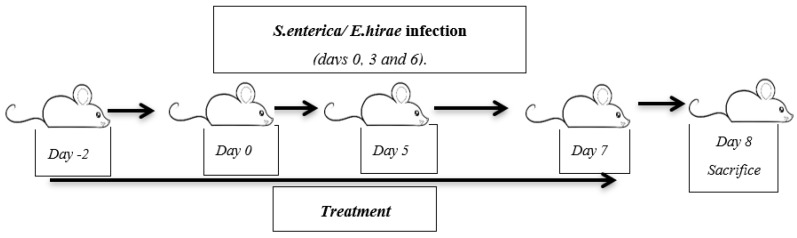
Schematic representation of the *S. enterica* and *E. hirae* infection model.

The induction of GE and UTI was made by two different infections by using *S. enterica* serovar Enteritidis (clinical isolate, phage type 4 according to international standards; B1241 culture of NIZO Food Research, Ede, The Netherlands) (2 × 10^9^ CFU/mL) and *E. hirae* Farrow and Collins (ATCC^®^ 8043™) (3 × 10^8^ CFU/mL) for 3 days (day 0, day 3, and day 6). The infections were induced by gavage administration. Two days before the bacterial administration, preventive oral treatments were performed by gavage every day until the seventh day [10,38,39] (Figure 9).

The virulence of this strain is sustained by routine oral passage in Sprague–Dawley rats, followed by isolation of translocated salmonella and enterococcus from the intestine and bladder. Salmonella and enterococcus were cultured and stored, as described previously [10,39,40]. At day 7 after infection, rats were killed by isoflurane inhalation.

Twenty-four hours after the last treatment, the animals were sacrificed for different analyses, in particular:

- Feces were collected for bacterial analysis.
- Urine was collected for pH measurement, bacterial counts, volume evaluation, and chemical analysis.
- Bladder and the low urethra were collected for primary cultures evaluating bacterial count.
- Intestine was collected for histological evaluation, myeloperoxidase assay, and immunostaining of tight junctions.

### 4.4. Experimental Groups

Rats were randomly allocated into the following groups:

(1) Control group (vehicle)—no *S. enterica*
(2) Control group (vehicle)—no *E. hirae*
(3) *S. enterica* group (2 × 10^9^ CFU/mL)
(4) *E. hirae* group (3 × 10^8^ CFU/mL)
(5) *S. enterica* + xyloglucan (10 mg/kg daily) + gelose (5 mg/kg daily)
(6) *E. hirae* + xyloglucan (10 mg/kg daily) + gelose (5 mg/kg daily)

The dose of xyloglucan + gelose was carried out using the previous dose-response experiments made in our laboratory.

### 4.5. Histological Examination

For histological examination, intestines were removed, fixed in 10% buffered formalin phosphate, embedded in paraffin sectioned, and stained with hematoxylin and eosin. The degree of inflammation on the microscopic cross-sections of the intestine was graded semi-quantitatively from 0 to 4 (0: no signs of inflammation, 1: very low level, 2: low level of leucocyte infiltration, 3: high level of leucocyte infiltration, high vascular density, and thickening of the colon wall, 4: transmural infiltration, loss of goblet cells, high vascular density, and thickening of the intestinal wall). Two experienced pathologists preformed grading in a blinded fashion by using an Olympus BX60 microscope (Olympus, Tokyo, Japan).

### 4.6. Myeloperoxidase Assay

Neutrophil infiltration in the intestine was monitored by measuring tissue MPO activity using a spectrophotometric assay with tetramethylbenzidine as substrate, according to a previously published method [41]. The rate of change in absorbance was measured spectrophotometrically at 650 nm. MPO activity was defined as the quantity of enzyme degrading 1 μmol of peroxide per min at 37 °C and was expressed in U/g wet tissue.

### 4.7. Immunohistochemical Localization of ZO-1 and Occludin

At 8 days after the *S. enterica* and *E. hirae* infections, intestinal tissues were fixed in 10% (*w*/*v*) PBS-buffered formaldehyde and 7 μm sections were prepared from paraffin embedded tissues. Immunohistochemical localization was performed as previously described [42]. Sections were incubated overnight with (1) purified goat polyclonal antibody directed towards ZO-1 (Santa Cruz Biotechnology, Santa Cruz, CA, USA, 1:500 in PBS, *v*/*v*) or (2) with purified hamster anti-Occludin (Santa Cruz Biotechnology, 1:500 in PBS, *w*/*v*). Sections were washed with PBS and incubated with secondary antibody. A biotin-conjugated goat anti-rabbit IgG and avidin–biotin peroxidase complex (Vector Laboratories, Burlingame, CA, USA) detected the specific labeling. The software Optilab Graftek (Graftek, Mirmande, France) assessed densitometry of immunohistochemistry photographs.

### 4.8. Bactericidal or Bacteriostatic Study

To evaluate bactericidal or bacteriostatic effect, we mixed 0.5 mL of xyloglucan + gelose with 0.5 mL of the *S. enterica* (2 × 10^9^ bacteria) *or E. hirae* inoculum (3 × 10^8^ bacteria). Then, 100 μL of this mixture were spread on Salmonella-Shigella (SS agar) and m-Enterococcus agar, respectively, and incubated at 37 °C overnight to assess bacterial growth.

The microbial growth was evaluated by colony-forming units (CFU) assay of the bacterial suspension, assuming that each colony has risen from one single bacterium. After incubation, colonies in the range 30–300 were considered to determine the number of CFU/mL, which was calculated as follows

$$\mathrm{CFU}=\frac{\left(\mathrm{number}\;\mathrm{of}\;\mathrm{colonies}\right)}{\mathrm{volume}\;\left(0.1\;\mathrm{mL}\right)\times \mathrm{dilution}\;\mathrm{factor}}$$

All values are given as mean ± SEM and are representative of at least 3 independent experiments. Data were examined by one-way analysis of variance followed by a Bonferroni post-hoc test for multiple comparisons. A *p* value of less than 0.05 was considered significant.
